# Mechanism of *Circ_HECW2* regulating osteoblast apoptosis in osteoporosis by attenuating the maturation of miR-1224-5p

**DOI:** 10.1186/s13018-023-04494-x

**Published:** 2024-01-06

**Authors:** Chao Zhang, Qiangqiang Li, Zhongduo Ye, Xiong Wang, Hui Zhao, Yongping Wang, Xingxing Zheng

**Affiliations:** 1 Department of Orthopedics, The First Hospital of Lanzhou University, Lanzhou, 73000 China; 2 Department of Ophthalmology, The Second Hospital of Lanzhou University, No. 82 Cuiyingmen, Chengguan District, Lanzhou, 730000 Gansu China

**Keywords:** Osteoporosis, Circ_HECW2, miR-1224-5p, Osteoblast, Apoptosis

## Abstract

**Background:**

Osteoporosis (OP) poses a significant clinical challenge with escalating morbidity. This study explores Circ_HECW2 expression in OP patients and its regulatory role in lipopolysaccharide (LPS)-induced osteoblast apoptosis.

**Methods:**

Circ_HECW2 expression in OP patient serum and healthy controls was quantified using RT-qPCR. Diagnostic value of Circ_HECW2 for OP was assessed via ROC curve. Pearson’s correlation model examined associations between indicators. Human osteoblasts HFOB1.19, treated with LPS, were analyzed for Circ_HECW2, pre-miR-1224, miR-1224-5p, and PDK2 mRNA levels. TUNEL assay determined cell apoptosis and Western blot assessed cleaved-caspase-3 protein levels. RNase R resistance assay and actinomycin D assay confirmed Circ_HECW2’s cyclic structure. RNA pull-down and dual-luciferase reporter assay verified binding relationships between Circ_HECW2 and miR-1224 and between miR-1224-5p and PDK2.

**Results:**

Circ_HECW2 exhibited elevated expression in OP patients with diagnostic significance and a negative correlation with lumbar T-score. LPS co-culture increased Circ_HECW2 expression in HFOB1.19 cells, significantly elevating apoptosis index and cleaved-caspase-3. Circ_HECW2 downregulation inhibited HFOB1.19 apoptosis, reduced pre-miR-1224 expression, and elevated mature miR-1224-5p. Circ_HECW2 bound to pre-miR-1224, and inhibiting miR-1224-5p reversed the effect of Circ_HECW2 downregulation on osteoblast apoptosis. miR-1224-5p targeted PDK2 transcription.

**Conclusion:**

Circ_HECW2, highly expressed in OP, holds diagnostic significance and reflects disease severity. Circ_HECW2 reduces mature miR-1224-5p by binding to pre-miR-1224, upregulating PDK2, and facilitating LPS-induced osteoblast apoptosis.

**Graphic Abstract:**

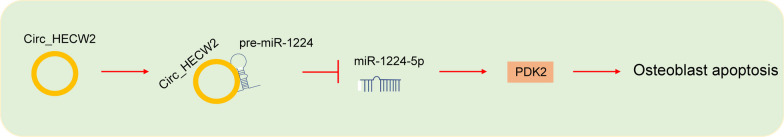

## Introduction

Osteoporosis (OP), an insidious metabolic skeletal disorder prevalent among the elderly and postmenopausal women, is characterized by diminished bone mass and degradation of bone microstructure, resulting in skeletal fragility and heightened fracture risks [1]. The pathogenesis of OP is intricate, and current understanding posits that OP primarily arises from an imbalance between bone formation and resorption, driven by dysfunctional osteoblast and osteoclast activities [2]. As the global population ages, OP emerges as a formidable threat to human health, escalating medical expenses costs, and imposing substantial burdens on OP patients and their families [3]. Compounding this issue is the absence of specific drugs in clinical practice for a definitive cure, with existing treatments only capable of delaying OP progression and mitigating fracture risks [4]. Consequently, there is an urgent need to identify novel and effective therapeutic targets for OP.

Circular RNAs (circRNAs) have emerged as a class of endogenous non-coding RNAs characterized by a unique covalent closed loop without a 5' cap or 3' tail. They play a crucial role in modulating various biological activities and pathophysiological responses [5]. Increasing studies have identified numerous deregulated circRNAs in bone metabolism, with circRNAs serving as potential diagnostic biomarkers for OP [6]. Circ_HECW2, for instance, exhibits an aberrantly high expression pattern in osteoarthritis patients and lipopolysaccharide (LPS)-exposed chondrocytes, such that Circ_HECW2 overexpression exacerbates LPS-induced chondrocyte apoptosis [7]. Additionally, Circ_HECW2 has been reported to be upregulated in the serum of coronary artery disease patients and oxidized low-density lipoprotein-induced human cardiac microvascular endothelial cells [8]. However, the regulatory mechanism of Circ_HECW2 in OP remains elusive.

Accumulating evidence highlights the presence of circRNA-miRNA networks in the intricate regulation of various bone diseases, including OP [9]. MicroRNAs (miRNAs), as non-coding small RNAs, intricately modulate gene expression post-transcriptionally, contributing significantly to the pathophysiology of diverse musculoskeletal conditions. For instance, specific miRNAs play pivotal roles in regulating cytokine expression, orchestrating the proliferation and differentiation of stromal cell lines involved in the composition of the extracellular matrix, thereby presenting therapeutic opportunities for managing tendon injuries [10]. miRNAs exhibit distinct expression patterns in osteoarthritis compared to nonosteoarthritic cartilage, and the complex interactions between miRNAs and their target genes are crucial for maintaining homeostatic pathways in osteoarthritis [11]. Notably, miRNAs are implicated in regulating the expression of OP-related genes and display abnormal expression profiles in OP patients [12]. Mechanistically, circRNAs function as molecular sponges for miRNAs, disrupting the expression of target messenger RNAs (mRNAs), a phenomenon substantiated in various bone cellular activities [13]. A deeper comprehension of the pivotal involvement of circRNA-miRNA-mRNA networks in OP may yield novel insights for exploring diagnostic biomarkers and therapeutic targets. Previous miRNome analysis has unveiled a reduction in miR-1224-5p expression in the serum samples of osteoporotic postmenopausal women [14]. Identified as a crucial osteogenic regulator, miR-1224-5p overexpression notably facilitates fracture healing and ameliorates OP progression resulting from estrogen deficiency and aging [15]. However, the regulatory network formed by Circ_HECW2 and miR-1224-5p in OP remains unexplored. This study endeavors to elucidate the expression pattern of Circ_HECW2 in OP and investigate its molecular mechanism in lipopolysaccharide (LPS)-induced osteoblast apoptosis, aiming to provide novel insights for the clinical management of OP.

## Materials and methods

### Ethics statement

This study received approval from the Ethics Committee of The First Hospital of Lanzhou University and adhered to the principles of the Declaration of Helsinki. Informed consent was obtained from all participants and their families.

### Study subjects

A total of 65 OP patients (OP group) who were diagnosed and treated at our hospital from July 2021 to July 2022 were enrolled, comprising 26 males and 39 females. Simultaneously, 65 healthy volunteers with normal bone mass were recruited as the control group, including 21 males and 44 females. Inclusion criteria encompassed patients diagnosed with OP by dual-energy x-ray absorptiometry (DEXA; Hologic Inc., Waltham, MA, US) (T-score ≤ − 2.5), capable of self-care in daily life, and able to communicate clearly without cognitive impairment. Exclusion criteria comprised patients with secondary OP caused by tumors, infections, and prolonged use of glucocorticoids; individuals with endocrine system diseases, neuro-muscular system diseases, blood diseases, and severe organ diseases; those who had taken drugs affecting bone metabolism within 3 months before seeking medical attention; and individuals mental disorders. Demographic details including age, gender, smoking history, body mass index (BMI), etc., were recorded. Blood samples were collected at admission and processed in Becton Dickinson (BD) ethylenediaminetetraacetic acid tubes, centrifuged at 3000×*g* for 15 min, and the serum was stored at − 80 °C for testing. Laboratory indicators such as white blood cells, parathyroid hormone (PTH), inorganic phosphorus, tP1NP, β-CTx, Calcium, 25(OH) D, Lymphocyte count, and Monocyte count were tested in strict accordance with operational procedures and kit instructions.

### Bone mineral density (BMD) evaluation

Patients, after discarding metal items, assumed a supine position at the center of the DEXA machine. Special pads were used to lift the lower limbs, aligning the hip and knee joints at a 90° angle. The initial laser point was positioned at the fifth lumbar spine. BMD values of the lumbar spine (L1, L2, L3, L4, and the sum of L1–L4), along with the *T* value by the system under big data, were collected. BMD values were measured meticulously by the same group of imaging physicians following technical specifications.

### Cell culture and treatment

Human osteoblasts HFOB1.19 (ATCC, Manassas, Virginia, USA; cells were identified through short tandem repeats, and Mycoplasma test result was negative) were cultured in medium containing 10% fetal bovine serum and 1% penicillin–streptomycin. The medium was refreshed every three days. Upon reaching 80% confluence, HFOB1.19 cells were subjected to co-culture with 10 µg/mL lipopolysaccharide (LPS; Solarbio, Beijing, China) added for 24 h. Cells without LPS treatment served as controls. Cell transfection was conducted before LPS addition. Briefly, HFOB1.19 cells in the logarithmic phase were transfected with si-Circ_HECW2 (si-NC) and anti-miR-1224-5p (anti-NC) (GenePharma Co., Ltd, Shanghai, China) according to the Lipofectamine 2000 reagent instructions (Thermo Fisher Scientific Inc., Waltham, MA, USA).

### Reverse transcription quantitative polymerase chain reaction (RT-qPCR)

Total RNA extracted from serum and osteoblasts using the TRIzol method (Beyotime, Shanghai, China) underwent reverse-transcribed into cDNA with the PrimeScript TM RT Reagent Kit (Takara, Tokyo, Japan). RT-qPCR was performed following the SYBR® Premix EX Taq TM II Kit instructions (Takara) on an ABI7500 PCR instrument (Invitrogen; Thermo Fisher Scientific), with cDNA as the template. The 2^−ΔΔCT^ method [16] calculated the relative expression of genes, using U6 as the internal reference of pre-miR-1224 and miR-1224-5p [17], and GAPDH as the internal reference of CircRNA and mRNA [18]. Primer sequences are provided in Table 1.

**Table 1:** qPCR primers

	Forward primer (5′–3′)	Reverse primer (5′–3′)
Circ_HECW2	CCCACCACTTTGAACGCTAC	GGCTGTCAATGCGTGCCT
HECW2	TACCACGGCATTAGTGGAGC	ATCCCTTTCTTTAGCCCAACTG
pre-miR-1224	TCGGCAGGGTGAGGACTCGGG	CAGCCACAAAAGAGCACAAT
miR-1224-5p	GCCGAGGTGAGGACTCGGGAGG	CTCAACTGGTGTCGTGGA
PDK2	CCCCGTCCCCGTTGTC	TCGCAGGCATTGCTGGAT
U6	CTCGCTTCGGCAGCACA	AACGCTTCACGAATTTGCGT
GAPDH	CGCTCTCTGCTCCTCCTGTTC	ATCCGTTGACTCCGACCTTCAC

PDK2: Pyruvate dehydrogenase kinase 2; GAPDH: glyceraldehyde-3-phosphate dehydrogenase

### Terminal deoxynucleotidyl transferase dUTP nick-end labeling (TUNEL)

Following transfection and LPS treatment, HFOB1.19 cells were prepared into smears, immersed in 40 g/L paraformaldehyde for 20 min, and then infiltrated with pre-cooled 1% Triton X-100. After sealing for 10 min, the cells were treated with the reaction solution per the TUNEL Cell Apoptosis Test Kit instructions (Beyotime), and finally added with the 4′,6-diamidino-2-phenylindole solution. TUNEL-positive cells were observed under a BX53 fluorescence microscope. The apoptosis index (number of apoptotic cells/number of cells in the field) was calculated based on counting TUNEL-positive cells that were counted in six randomly selected fields of view.

### Western blot

Cell protein was extracted using radio-immunoprecipitation assay lysis buffer (Beyotime), and the protein determined was detected using a bicinchoninic acid kit (Beyotime). Equivalent amounts of protein were separated by 12% SDS-PAGE (Beyotime) and transferred onto polyvinylidene fluoride membranes by electrophoresis. After blocking, the membranes were incubated with primary antibodies cleaved-caspase-3 (ab2302, 1:50, Abcam, Cambridge, MA, USA) and GAPDH (ab128915, 1:10,000, Abcam). Following rinsing, the membranes were further incubated with the corresponding secondary antibody (ab205718, 1:2000, Abcam) in the dark for 2 h. After another round of rinsing, the enhanced chemiluminescence assay kit was used for imaging, and Bandscan 5.0 facilitated the quantitative detection of protein bands.

### Ribonuclease R (RNase R) resistance assay

Total RNA was extracted from HFOB1.19 cells. Subsequently, 1.5 μg of RNA was digested with or without RNase R (Thermo Fisher Scientific) for 10 min. After reverse transcription, RT-qPCR was performed to detect the mRNA levels of Circ_HECW2 and linear HECW2.

### Actinomycin D treatment

HFOB1.19 cells underwent treatment with 1 μg/mL actinomycin D for different time points (0, 8, 16, and 24 h). Total RNA was then extracted for RT-qPCR to assess the mRNA levels of Circ_HECW2 and linear HECW2.

### Subcellular fractionation

HFOB1.19 cells were subjected to an ice bath in cytoplasmic lysate for 10 min and then centrifuged to separate the cytoplasmic supernatant from the cell particles containing the nucleus. RNA was subsequently obtained from cytoplasmic and nuclear extracts using the PARIS reagent kit (Invitrogen). RT-qPCR determined the levels of Circ_HECW2, U6 (nuclear control), and GAPDH (cytoplasmic control).

### Bioinformatics

The binding site between Circ_HECW2 and pre-miR-1224 was predicted using the IntaRNA website (https://rna.informatik.uni-freiburg.de/) [19]. Downstream target genes of miR-1224-5p and corresponding binding sites were predicted and screened through TargetScan7.2 (http://www.targetscan.org/vert_72/) [20] and miRDB (http://mirdb.org/) databases [21].

### Dual-luciferase reporter gene assay

The binding sites between Circ_HECW2 and pre-miR-1224 and between miR-1224-5p and PDK2 were predicted through the IntaRNA and TargetScan7.2 databases, respectively. Synthesized Circ_HECW2-WT, containing the pre-miR-1224 binding site, and Circ_HECW2-MUT, mutated according to the binding site, as well as the PDK2 3'UTR fragment containing the miR-1224-5p binding site (PDK2-WT) and PDK2-MUT, were individually constructed into the dual-luciferase report vectors (LMAI, shanghai, China). These plasmids were co-transfected with mimic-NC, pre-miR-1224-mimic, and miR-1224-5p-mimic into HFOB1.19 cells using Lipofectamine 2000. After 48 h, the cells were collected and analyzed on the dual-luciferase reporter detection system (Promega, Madison, WI, USA).

### RNA pull-down

Biotinylated miR-NC, pre-miR-1224-WT, and pre-miR-1224-MUT (Invitrogen) were transfected into HFOB1.19 cells using Lipofectamine 2000. Following ultrasound treatment, cell lysates were collected and incubated with Dynabeads streptomyces avidin magnetic beads M-280 (Thermo Fisher Scientific). The bound RNA was purified using TRIzol reagent, and the enrichment of Circ_HECW2 was measured using RT-qPCR.

### Statistical analysis

Data analysis and graphical representation were performed using the SPSS 22.0 (IBM Corp., Armonk, NY, USA) and GraphPad Prism 8.0 (GraphPad Software Inc., San Diego, CA, USA). Counting data were expressed as percentages, and comparisons between two groups were conducted using the *χ*^2^ test. Measurement data were presented as mean ± standard deviation. The *t* test was employed for comparisons between two groups. One-way or two-way analysis of variance (ANOVA) was used for comparisons among multiple groups, followed by Tukey’s multiple comparison test. The receiver operating characteristic curve (ROC) was utilized to analyze the diagnostic efficiency of Circ_HECW2 in OP. Pearson’s correlation coefficient was applied to analyze the correlation of serum Circ_HECW2 and serum pre-miR-1224 with mature miR-1224-5p, as well as the correlation of serum miR-1224-5p with PDK2 mRNA. A significance level of *P* < 0.05 was considered statistically significant.

## Results

### Circ_HECW2 was highly expressed in OP patients; Circ_HECW2 had diagnostic value for OP and was related to disease characteristics

**Fig. 1:**
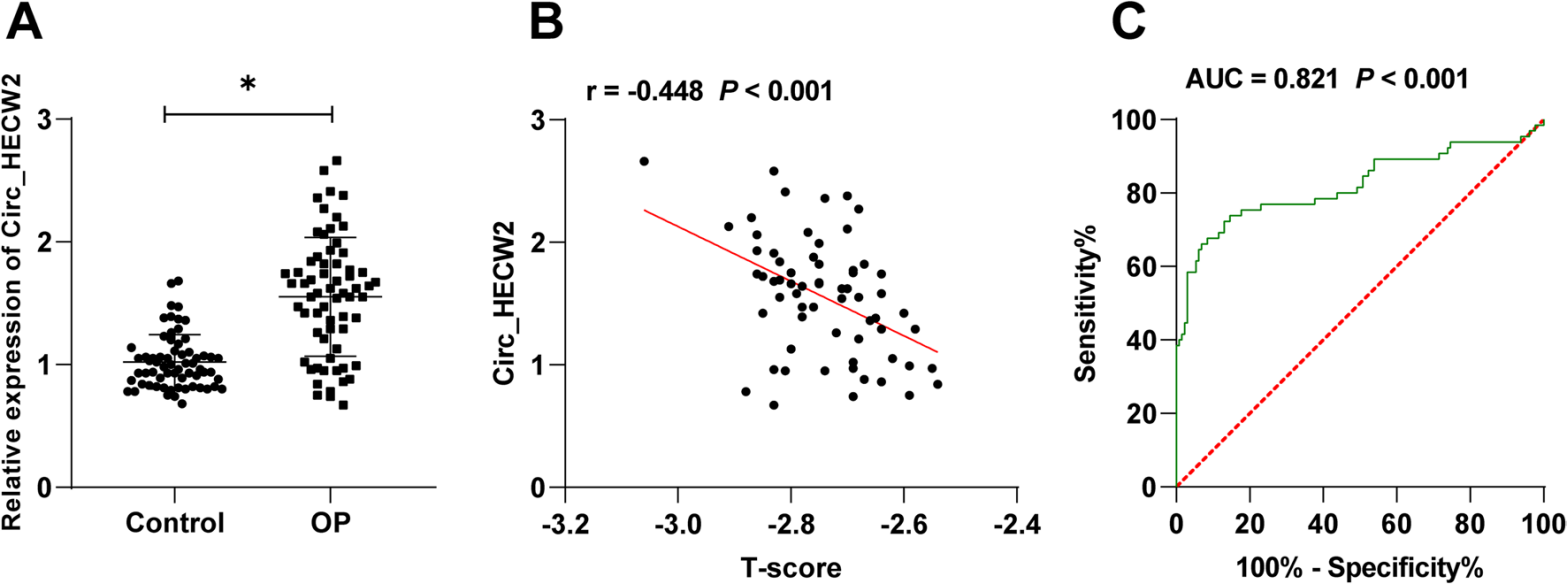
Circ_HECW2 was highly expressed in OP patients; Circ_HECW2 had diagnostic value for OP and was related to disease characteristics. Serum samples from healthy controls and OP patients. **A** RT-qPCR detected Circ_HECW2 expression in serum. **B** Pearson's correlation analyzed the correlation between Circ_HECW2 and lumbar spine (L1–L4) T-score. **C** ROC curve analyzed the diagnostic effectiveness of Circ_HECW2 for OP. Control: *n* = 65; OP: *n* = 65. Data in panel **A** were analyzed using *t* test, *P < 0.05

Clinical characteristics were collected from OP patients and healthy volunteers (Table 2). No significant differences were observed in age, gender, BMI, and various biochemical indicators [PTH, tP1NP, calcium, inorganic phosphorus, 25(OH) D, WBC, lymphocyte count, and monocyte count] between the OP and control groups. However, OP patients exhibited significantly lower BMD and lung spine (L1–L4) T-scores and higher β-CTx than the control group (*P* < 0.05). Investigation of Circ_HECW2 in the serum revealed its heightened expression in OP patients (*P* < 0.05, Fig. 1A), correlating negatively with the T-score (*P* < 0.05, Fig. 1B). ROC analysis demonstrated Circ_HECW2 had diagnostic value for OP (AUC = 0.821). When Circ_HECW2 > 1.23, sensitivity was 73.85%, and specificity was 84.62% (*P* < 0.05, Fig. 1C).

**Table 2:** Clinical characteristics of patients

Data	Control (*n* = 65)	OP (*n* = 65)	*P* value
Age (years)	56.89 ± 5.99	56.03 ± 6.44	0.431
Gender (Male/Female)	26/39	21/44	0.361
BMI (kg/m^2^)	22.89 ± 2.54	23.14 ± 2.39	0.564
BMD (g/cm^2^)	0.89 ± 0.09	0.53 ± 0.06	< 0.001
Lumbar spine (L1–L4) T-score	− 0.24 ± 0.03	− 2.74 ± 0.10	< 0.001
PTH (ng/L)	43.49 ± 14.65	40.49 ± 11.50	0.197
β-CTx (ng/L)	359.72 ± 112.97	425.61 ± 138.38	0.004
tP1NP (ng/L)	44.24 ± 9.97	45.66 ± 11.28	0.449
Calcium (mmol/L)	2.27 ± 0.14	2.28 ± 0.15	0.693
Inorganic phosphorus (mmol/L)	1.20 ± 0.08	1.17 ± 0.10	0.063
25(OH) D (ng/ml)	22.04 ± 5.81	23.16 ± 5.10	0.243
WBC (10^9^/L)	6.40 ± 0.51	6.38 ± 0.40	0.795
Lymphocyte count (10^9^/L)	2.24 ± 0.18	2.26 ± 0.14	0.470
Monocyte count (10^9^/L)	0.49 ± 0.03	0.50 ± 0.04	0.113

BMI: Body mass index; BMD: bone mineral density; PTH: parathyroid hormone; β-CTx: beta-isomer of the C-terminal telopeptide of type I collagen; tP1NP: total procollagen I N-terminal propeptide; 25(OH) D: 25-hydroxyvitamin D; WBC: white blood counts. The data of age, BMI, BMD, lumbar spine (L1–L4) T-score, PTH, β-CTx, tP1NP, calcium, inorganic phosphorus, 25(OH) D, WBC, lymphocyte count, and monocyte count were expressed as mean ± standard deviation and subjected to *t* test. The gender data were represented by number of cases and subjected to *χ*^2^ test

### Circ_HECW2 was highly expressed in LPS-induced osteoblasts

**Fig. 2:**
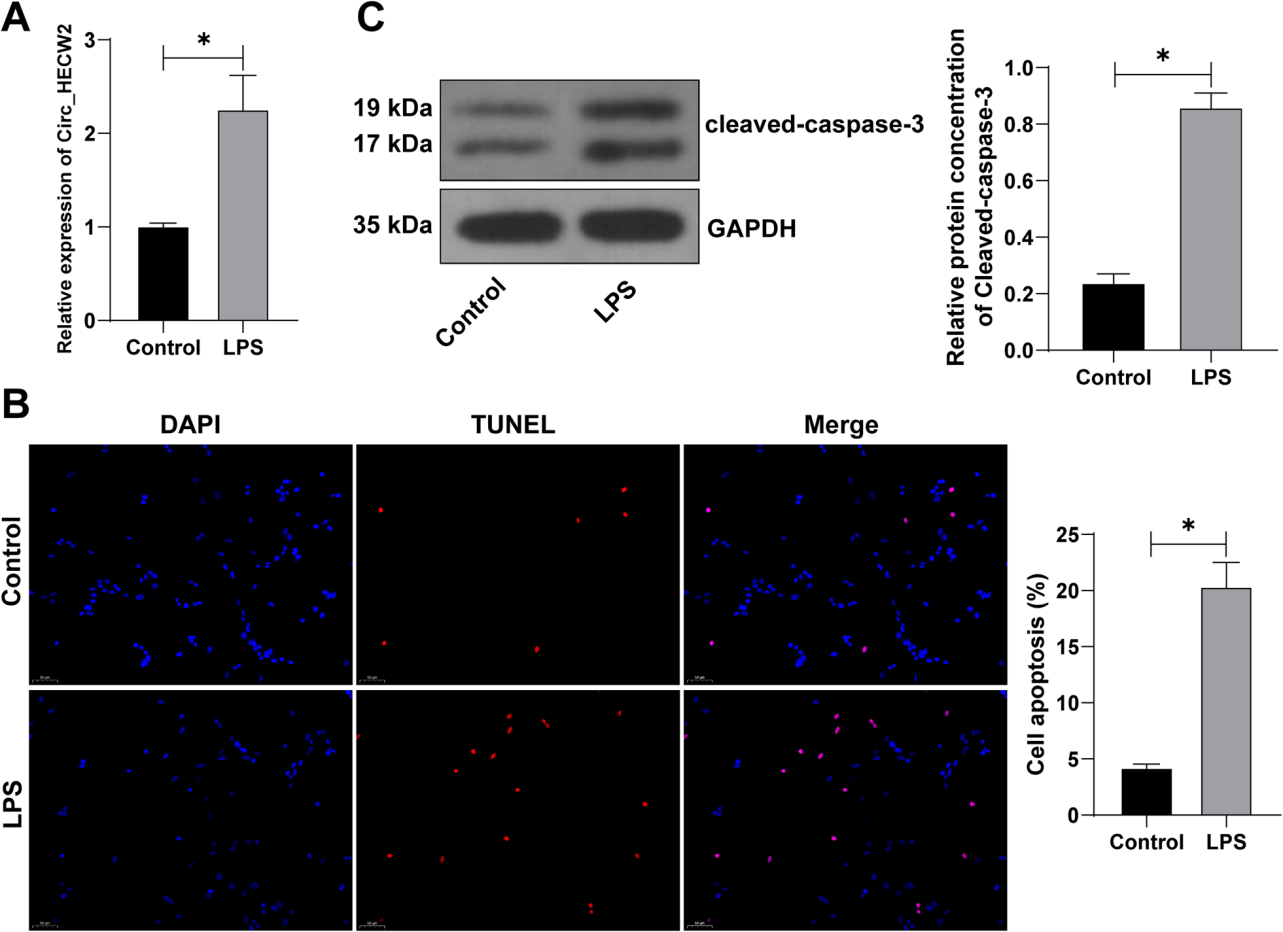
Circ_HECW2 was highly expressed in LPS-induced osteoblasts. Human osteoblasts HFOB1.19 were treated with 10 µg/mL LPS for 24 h, with cells free of LPS treatment as the control. **A** RT-qPCR detected Circ_HECW2 expression in cells. **B** TUNEL detected cell apoptosis. **C** Western blot detected the apoptosis related protein cleaved-caspase-3. The cell experiments were independently repeated 3 times. Data were expressed as mean ± standard deviation and analyzed using *t* test, *P < 0.05

Given LPS's influence on Circ_HECW2 expression and its promotion of cell apoptosis and inflammatory responses [7], we co-cultured LPS with HFOB1.19 cells. This resulted in a significant increase in the apoptosis index, cleaved-caspase-3 protein (*P* < 0.05, Fig. 2B, C), and Circ_HECW2 expression in HFOB1.19 cells (*P* < 0.05, Fig. 2A). These findings indicated that LPS induced osteoblast apoptosis, potentially through the upregulation of HECW2.

### Downregulation of Circ_HECW2 suppressed LPS-induced osteoblast apoptosis

**Fig. 3:**
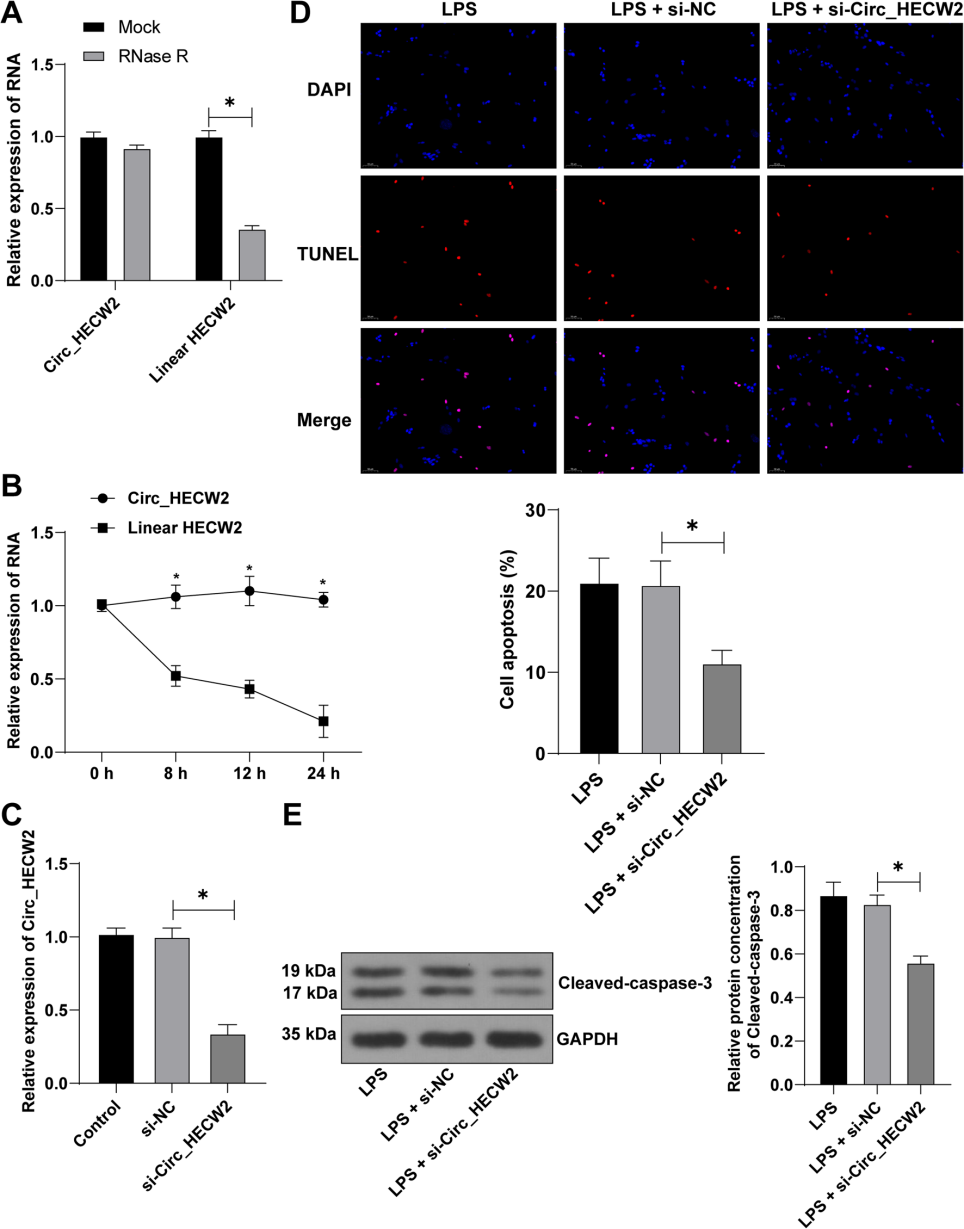
Downregulation of Circ_HECW2 suppressed LPS-induced osteoblast apoptosis. Before LPS treatment, HFOB1.19 cells were transfected with si-Circ_HECW2, with si-NC as the control. A-B: RNase R enzyme digestion experiment (**A**) and actinomycin D experiment (**B**) determined the circular structure of Circ_HECW2. **C** RT-qPCR detected Circ_HECW2 expression in cells. **D** TUNEL detected cell apoptosis. **E** Western blot detected the apoptosis related protein cleaved-caspase-3. The cell experiments were independently repeated 3 times. Data in panels AB were analyzed using two-way ANOVA, and data in panels C/D/E were analyzed using one-way ANOVA, followed by Tukey's multiple comparisons test, *P < 0.05

To confirm Circ_HECW2’s role in HFOB1.19 cell apoptosis, we verified its circular structure through RNase R enzyme digestion and actinomycin D experiments (Fig. 3A, B). Preceding LPS treatment, HFOB1.19 cells were transfected with si-Circ_HECW2 (*P* < 0.05, Fig. 3C). Downregulating Circ_HECW2 expression significantly reduced the apoptosis index and cleaved-caspase-3 protein (*P* < 0.05, Fig. 3D, E), underscoring the regulatory role of Circ_HECW2 in HFOB1.19 cell apoptosis.

### Circ_HECW2 reduced the formation of mature miR-1224-5p by binding to pre-miR-1224

**Fig. 4:**
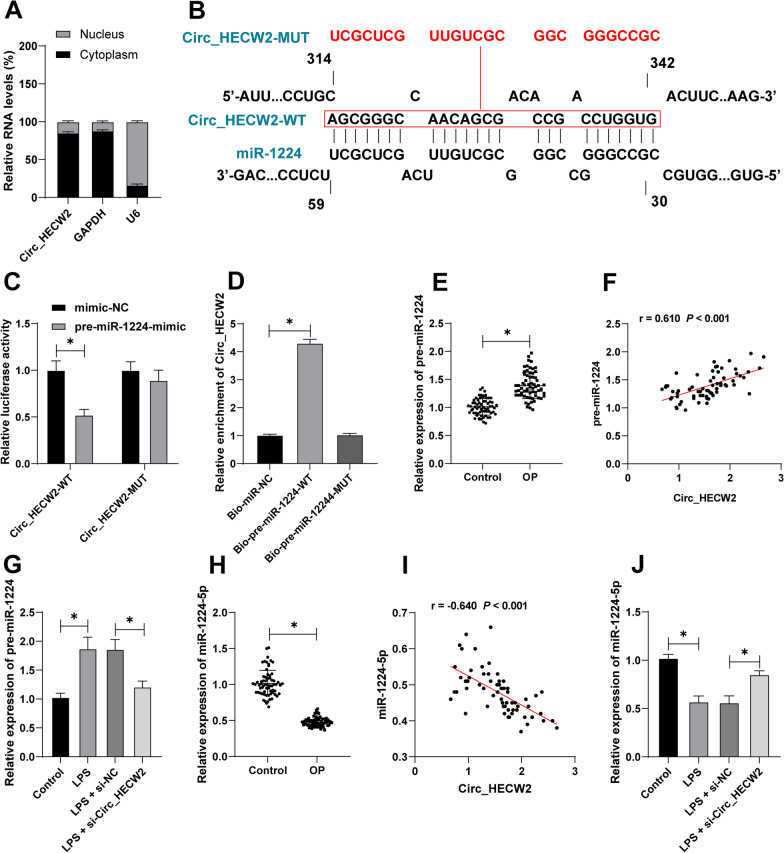
Circ_HECW2 reduced the formation of mature miR-1224-5p by binding to pre-miR-1224. **A** Subcellular fractionation experiment determined Circ_HECW2 distribution in HFOB1.19 cells. **B** The binding site between Circ_HECW2 and pre-miR-1224 was predicted on the IntaRNA website. **C** Dual-luciferase reporter gene experiment determined the binding relationship between Circ_HECW2 and pre-miR-1224. **D** RNA pull-down detected the enrichment of Circ_HECW2 on the binding site of pre-miR-1224. **E** RT-qPCR detected pre-miR-1224 expression in serum. **F** Correlation analysis between serum Circ_HECW2 and pre-miR-1224 in OP patients. **G** RT-qPCR detected pre-miR-1224 expression in HFOB1.19 cells. **H** RT-qPCR detected miR-1224-5p expression in serum. **I** Correlation analysis between serum Circ_HECW2 and miR-1224-5p in OP patients. **J** RT-qPCR detected miR-1224-5p expression in HFOB1.19 cells. The cell experiments in panels **A**/**C**/**D**/**G**/**J** were independently repeated 3 times. **E**, **H** Control, *n* = 65; OP, *n* = 65. Data in panels **E**/**H** were analyzed using independent *t* test. Data in panel **C** were analyzed using two-way ANOVA, and data in panels **D**/**G**/**J** were analyzed using one-way ANOVA, followed by Tukey's multiple comparisons test, *P < 0.05

To decipher the molecular underpinnings of Circ_HECW2 in osteoblast apoptosis, we initiated investigations into its interaction with miR-1224-5p. Subcellular fractionation experiments highlighted the predominant cytoplasmic expression of Circ_HECW2 (Fig. 4A), suggesting its potential role as a competing endogenous RNA (ceRNA). Notably, miR-1224-5p overexpression has been associated with the attenuation of OP progression [15], and the inhibition of pre-miR-1224 has been linked to a reduction in osteoclast generation [22]. Computational prediction using the IntaRNA website identified a binding site between Circ_HECW2 and pre-miR-1224 (Fig. 4B). This interaction was substantiated through dual-luciferase reporter gene assays and RNA pull-down experiments (*P* < 0.05, Fig. 4C, D).

Further investigation into the serum of study subjects unveiled elevated expression of pre-miR-1224 in OP patients, positively-correlated with Circ_HECW2 expression (*P* < 0.05, Fig. 4E, F). Conversely, miR-1224-5p exhibited lower expression in OP patients and displayed a negative correlation with Circ_HECW2 (*P* < 0.05, Fig. 4H, I). In LPS-treated HFOB1.19 cells, high pre-miR-1224 and low miR-1224-5p expressions were observed. Notably, downregulating Circ_HECW2 decreased pre-miR-1224 but increased miR-1224-5p expression (*P* < 0.05, Fig. 4G, J).

Collectively, these findings suggest that Circ_HECW2 may impede the formation of mature miR-1224-5p by competitively-binding to pre-miR-1224.

### miR-1224-5p inhibition reversed the inhibitory effect of HECW2 downregulation on osteoblast apoptosis

**Fig. 5:**
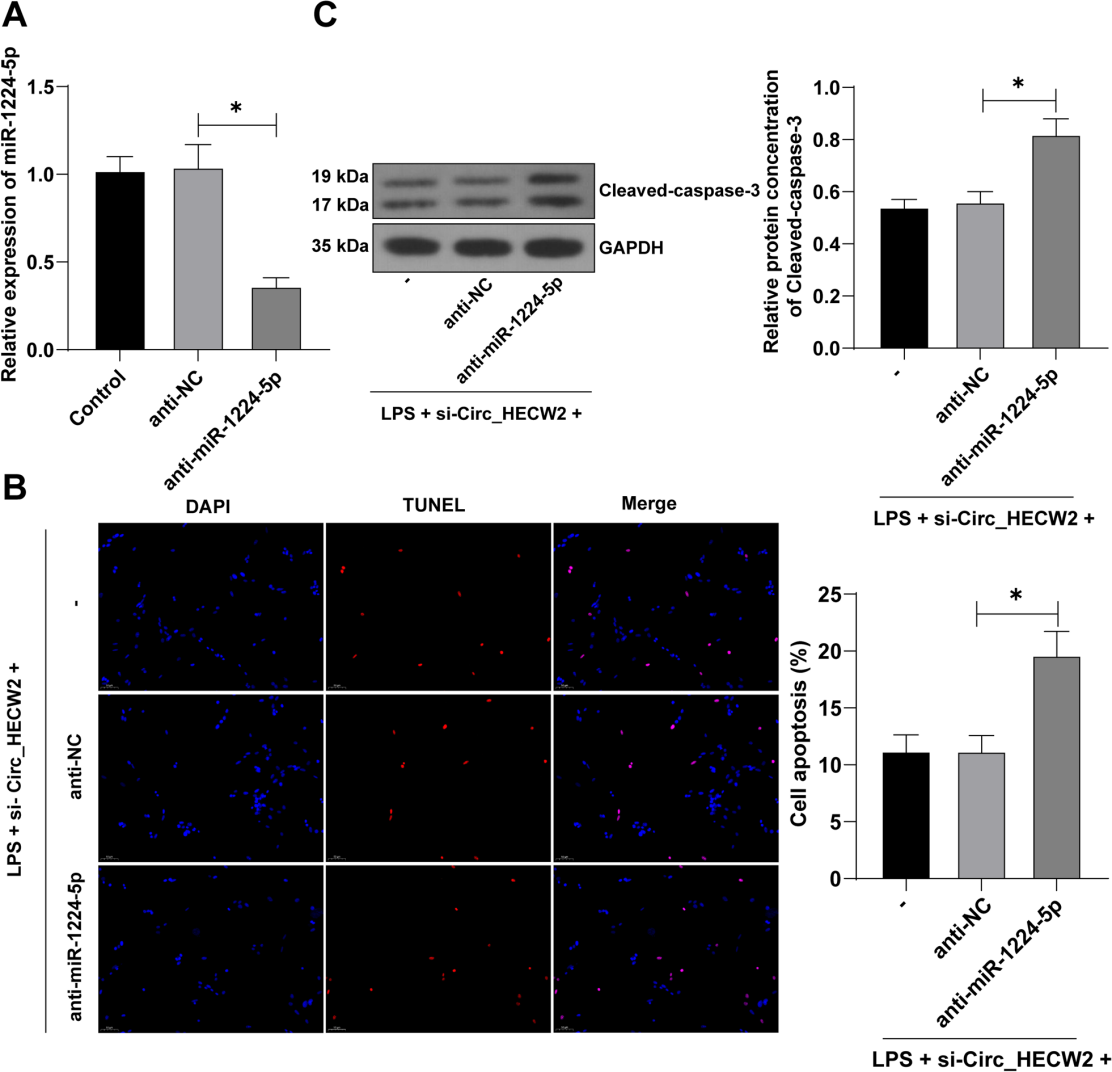
miR-1224-5p inhibition reversed the inhibitory effect of HECW2 downregulation on osteoblast apoptosis. Before LPS treatment, HFOB1.19 cells were transfected with anti-miR-1224-5p, with anti-NC as the control, followed by combined treatment with si-Circ_HECW2. **A** RT-qPCR detected miR-1224-5p expression in cells. **B** TUNEL detected cell apoptosis. **C** Western blot detected the apoptosis related protein cleaved-caspase-3. The cell experiments were independently repeated 3 times. Data were analyzed using one-way ANOVA, followed by Tukey's multiple comparisons test, *P < 0.05

To verify the role of Circ_HECW2 and miR-1224-5p in regulating HFOB1.19 cell apoptosis, HFOB1.19 cells were transfected with anti-miR-1224-5p (*P* < 0.05, Fig. 5A) and subjected to a combined treatment with si-Circ_HECW2. Compared with transfection of si-Circ_HECW2 alone, the combined transfection of si-Circ_HECW2 and anti-miR-1224-5p significantly augmented the apoptosis index and cleaved-caspase-3 protein (*P* < 0.05, Fig. 5B, C). These results indicate that the reduction of mature miR-1224-5p counteracted the inhibitory effect of Circ_HECW2 silencing on osteoblast apoptosis.

### PDK2 was a downstream target gene of miR-1224-5p and was highly expressed in OP patients

**Fig. 6:**
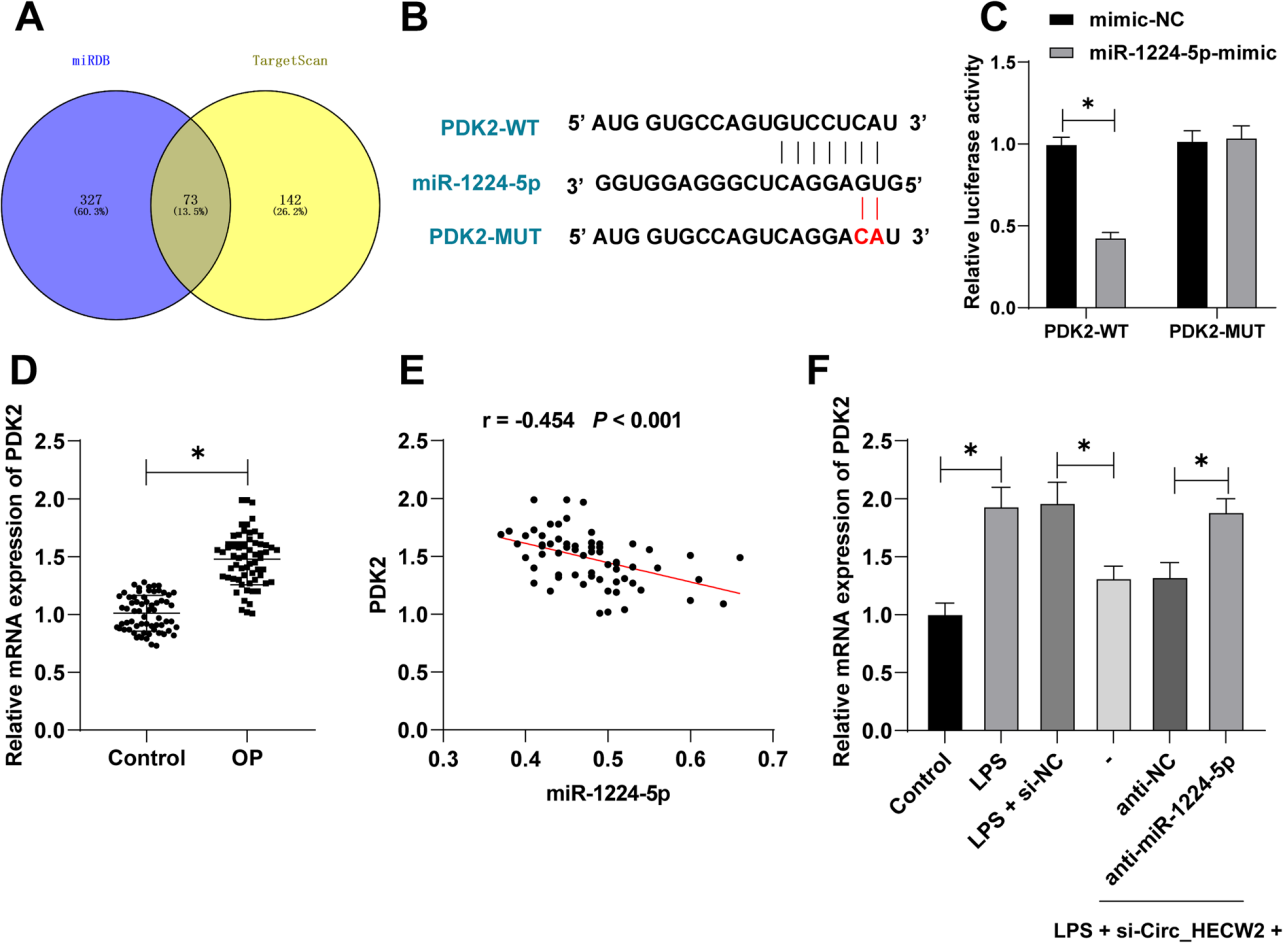
PDK2 was a downstream target gene of miR-1224-5p and was highly expressed in OP patients. **A** The downstream target genes of miR-1224-5p were predicted through the TargetScan7.2 and miRDB databases. **B** The binding site of miR-1224-5p and PDK2 was predicted through the TargetScan7.2 database. **C** Dual-luciferase reporter gene experiment determined the binding relationship between miR-1224-5p and PDK2. **D** RT-qPCR detected PDK2 mRNA expression in serum. **E** Correlation analysis between serum miR-1224-5p and PDK2. **F** RT-qPCR detected PDK2 mRNA expression in cells. The cell experiments in panels **C**/**F** were independently repeated 3 times. **D** Control, *n* = 65; OP, *n* = 65. Data in panel D were analyzed using independent *t* test. Data in panel **C** were analyzed using two-way ANOVA, and data in panel **F** were analyzed using one-way ANOVA, followed by Tukey's multiple comparisons test, *P < 0.05

In our pursuit to unravel downstream targets of miR-1224-5p, we utilized TargetScan7.2 and miRDB, revealing PDK2 as a potential target (Fig. 6A). PDK2 inhibition has demonstrated efficacy in ameliorating OP by mitigating the activation of aberrant osteoclasts [23]. TargetScan7.2 predictions indicated a binding site between miR-1224-5p and PDK2 (Fig. 6B), and this interaction was validated through dual-luciferase reporter gene assays (*P* < 0.05, Fig. 6C).

Further examination of serum samples from OP patients showed a significant elevation in PDK2 mRNA expression compared to healthy controls (*P* < 0.05, Fig. 6D). Additionally, a substantial negative correlation was observed between PDK2 mRNA expression and miR-1224-5p levels (*P* < 0.05, Fig. 6E).

Upon Circ_HECW2 downregulation, PDK2 mRNA expression in cells significantly decreased. Notably, the combined treatment, compared to si-Circ_HECW2 treatment alone, markedly increased the PDK2 expression in cells (*P* < 0.05, Fig. 6F). These findings suggested that Circ_HECW2 potentially upregulates PDK2 expression by suppressing the formation of mature miR-1224-5p, thereby promoting osteoblast apoptosis.

## Discussion

In the ever-expanding landscape of high-throughput RNA sequencing studies, the aberrant expression of circRNAs in OP has come to the forefront [6]. In a pioneering contribution, our study underscores the heightened expression of Circ_HECW2 in OP patients, establishing its diagnostic significance for the condition. Specifically, Circ_HECW2’s involvement in accelerating LPS-induced osteoblast apoptosis is delineated. This acceleration is attributed to Circ_HECW2's competitive binding to pre-miR-1224, thereby diminishing the formation of mature miR-1224-5p and orchestrating the upregulation of PDK2 expression.

The intricate interplay between osteoclast-mediated bone resorption and osteoblast-mediated bone formation is a hallmark of OP pathogenesis, disrupting the delicate balance of bone remodeling and precipitating the onset of OP [24]. Programmed cell death mechanisms, such as apoptosis, ferroptosis, and pyroptosis, play a decisive role in bone cell fate and exert a profound impact on bone metabolism [25]. Within this context, osteoblast apoptosis emerges as a pivotal event in the initiation and progression of OP, positioning the inhibition of osteoblast apoptosis as a promising avenue for OP treatment [2]. In our exploration of Circ_HECW2's regulatory mechanism in OP, we opted for LPS induction to simulate osteoblast apoptosis. Subsequent to LPS treatment, both the apoptosis index and cleaved-caspase-3 protein witnessed a significant surge. Notably, there was a conspicuous rise in Circ_HECW2 expression in HFOB1.19 cells following LPS exposure. This aligns with previous findings where Circ_HECW2 exhibited significant upregulation in osteoarthritis patients and LPS-exposed chondrocytes, and its overexpression exacerbated LPS-triggered chondrocyte apoptosis [7]. Moreover, Circ_HECW2 silencing has been demonstrated to impede LPS-triggered endothelial–mesenchymal transition and cell apoptosis in human brain microvascular endothelial cells [26]. siRNAs are useful to identify molecular targets and evaluate the efficacy of specific drugs and can themselves be used for therapeutic purposes in tendon healing, rheumatoid arthritis, and OP [27–29]. The transfection of si-Circ_HECW2 in our study corroborated these trends, significantly reducing the apoptosis index and cleaved-caspase-3 protein in HFOB1.19 cells. This lends credence to the notion that Circ_HECW2 downregulation exerts a suppressive effect on osteoblast apoptosis in OP.

Subsequently, our focus shifted to unraveling the intricate molecular mechanism through which Circ_HECW2 regulates osteoblast apoptosis. CircRNAs exert a profound influence on diverse physiological and pathological processes by modulating gene transcription and translation, mediating alternative splicing, or acting as miRNA sponges in specific tissues [9]. For instance, in a prior study, Circ_HECW2 was identified as a miR-30e-5p sponge, enhancing endothelial–mesenchymal transition triggered by LPS [26]. Our investigation confirmed that Circ_HECW2 is predominantly expressed in the cytoplasm, suggesting its potential role as a mediator of osteoblast apoptosis by functioning as a miRNA sponge. Dysregulation of miRNA-mediated mechanisms stands out as a prominent pathological factor in OP [30]. Upon literature review, the significant role of miR-1224-5p in osteoclast responses and proliferation drew our attention [22]. Exosomal miR-1224 expedites osteoclast differentiation in patients with brain trauma via the Hippo signaling pathway [31]. Canonical miRNA biogenesis undergoes a two-step process: from primary transcripts (pri-miRNAs) to pre-miRNAs and from pre-miRNAs to mature miRNAs, mediated by DROSHA and DICER, respectively [32]. IntaRNA website prediction unveiled a binding site between Circ_HECW2 and pre-miR-1224, a relationship validated through dual-luciferase reporter gene assay and RNA pull-down assays. Pre-miR-1224 exhibited heightened expression, while miR-1224-5p was poorly expressed in OP patients and LPS-treated HFOB1.19 cells. Downregulating Circ_HECW2 reduced the expression of pre-miR-1224 but elevated the expression of miR-1224-5p. We postulate that Circ_HECW2 might diminish the formation of mature miR-1224-5p by competitively-binding to pre-miR-1224. Notably, miRNA-1224-5p knockdown impedes osteoblast differentiation, suppresses bone regeneration, and accelerates OP progression in mouse models [15]. Consistent with these findings, we observed that miR-1224-5p inhibition reversed the inhibitory effect of HECW2 downregulation on osteoblast apoptosis, signifying that Circ_HECW2 promotes osteoblast apoptosis by reducing the formation of mature miR-1224-5p.

Finally, leveraging TargetScan7.2 and miRDB databases, we conducted a screening to identify the downstream genes of miR-1224-5p. Pyruvate dehydrogenase kinases (PDKs), a group of gatekeeper enzymes, play a pivotal role in metabolic regulation by modulating the activity of pyruvate dehydrogenase complex [33]. Among the four PDK isoenzymes discovered in mammalian tissues, PDK2 inhibition has been shown to prevent ovariectomy-induced bone loss during osteoclastogenesis in mice by suppressing aberrant osteoclast activation [23]. Our results confirmed the binding relationship between miR-1224-5p and PDK2. Moreover, PDK2 mRNA expression was significantly elevated in the serum of OP patients, and downregulation of Circ_HECW2 reduced PDK2 mRNA expression in LPS-treated HFOB1.19 cells. These findings suggest that Circ_HECW2 might upregulate PDK2 expression by repressing the formation of mature miR-1224-5p, thereby fostering osteoblast apoptosis.

## Conclusion

In summary, our findings underscore the diagnostic significance the heightened expression of Circ_HECW2 in osteoporosis (OP), shedding light on its role in fostering osteoclast apoptosis through the intricate miR-1224-5p/PDK2 axis. Despite the valuable insights gained, this study encounters certain limitations. The exploration of Circ_HECW2’s regulatory impact on OP remains confined to the cellular level, warranting future investigations involving animal models to validate its mechanisms. The specificity of Circ_HECW2 in regulating additional targets within the context of OP remains unresolved. Furthermore, the downstream target gene of miR-1224-5p, PDK2, merits further exploration, extending our comprehension of the intricate molecular network involved in OP pathogenesis. Future endeavors will encompass the verification of Circ_HECW2's regulatory mechanism in vivo, coupled with a more in-depth analysis of PDK2, to advance our understanding of the comprehensive regulatory landscape in osteoporosis.

## Data Availability

The datasets used and analyzed during the current study are available from the corresponding author upon reasonable request.

## Abbreviations

OP: Osteoporosis
circRNA: Circular RNA
PDK2: Pyruvate dehydrogenase kinase 2
LPS: Lipopolysaccharide
miRNAs: MicroRNAs
BMI: Body mass index
PTH: Parathyroid hormone
BMD: Bone mineral density
RT-qPCR: Reverse transcription quantitative polymerase chain reaction
TUNEL: Terminal deoxynucleotidyl transferase dUTP nick-end labeling
RNase R: Ribonuclease R
ROC: Receiver operating characteristic curve
GAPDH: Glyceraldehyde-3-phosphate dehydrogenase
ANOVA: Analysis of variance

## References

[1] Rachner TD, Khosla S, Hofbauer LC (2011). Osteoporosis: now and the future. Lancet.

[2] Chen X, Wang Z, Duan N, Zhu G, Schwarz EM, Xie C (2018). Osteoblast-osteoclast interactions. Connect Tissue Res.

[3] Boschitsch EP, Durchschlag E, Dimai HP (2017). Age-related prevalence of osteoporosis and fragility fractures: real-world data from an Austrian Menopause and Osteoporosis Clinic. Climacteric.

[4] Aibar-Almazan A, Voltes-Martinez A, Castellote-Caballero Y, Afanador-Restrepo DF, Carcelen-Fraile MDC, Lopez-Ruiz E (2022). Current status of the diagnosis and management of osteoporosis. Int J Mol Sci.

[5] Moura SR, Fernandes MJ, Santos SG, Almeida MI (2023). Circular RNAs: promising targets in osteoporosis. Curr Osteoporos Rep.

[6] Chen W, Zhang B, Chang X (2021). Emerging roles of circular RNAs in osteoporosis. J Cell Mol Med.

[7] Zuo J, Chen C, Zhang X, Wu J, Li C, Huang S (2021). Circ_HECW2 regulates LPS-induced apoptosis of chondrocytes via miR-93 methylation. Immun Inflamm Dis.

[8] Wei W, Tang M, Wang Q, Li X (2022). Circ_HECW2 regulates ox-LDL-induced dysfunction of cardiovascular endothelial cells by miR-942-5p/TLR4 axis. Clin Hemorheol Microcirc.

[9] Huang W, Wu Y, Qiao M, Xie Z, Cen X, Huang X (2022). CircRNA-miRNA networks in regulating bone disease. J Cell Physiol.

[10] Giordano L, Porta GD, Peretti GM, Maffulli N (2020). Therapeutic potential of microRNA in tendon injuries. Br Med Bull.

[11] Oliviero A, Della Porta G, Peretti GM, Maffulli N (2019). MicroRNA in osteoarthritis: physiopathology, diagnosis and therapeutic challenge. Br Med Bull.

[12] Lu W, Wang Q, Xue Y, Gu J, Yao P, Ge Y (2021). Identification of potential osteoporosis miRNA biomarkers using bioinformatics approaches. Comput Math Methods Med.

[13] Gao M, Zhang Z, Sun J, Li B, Li Y (2022). The roles of circRNA-miRNA-mRNA networks in the development and treatment of osteoporosis. Front Endocrinol (Lausanne).

[14] Pepe J, Rossi M, Battafarano G, Vernocchi P, Conte F, Marzano V (2022). Characterization of extracellular vesicles in osteoporotic patients compared to osteopenic and healthy controls. J Bone Miner Res.

[15] Hu L, Xie X, Xue H, Wang T, Panayi AC, Lin Z (2022). MiR-1224-5p modulates osteogenesis by coordinating osteoblast/osteoclast differentiation via the Rap1 signaling target ADCY2. Exp Mol Med.

[16] Livak KJ, Schmittgen TD (2001). Analysis of relative gene expression data using real-time quantitative PCR and the 2(-Delta Delta C(T)) method. Methods.

[17] Li S, Shi Z, Fu S, Li Q, Li B, Sang L (2021). Exosomal-mediated transfer of APCDD1L-AS1 induces 5-fluorouracil resistance in oral squamous cell carcinoma via miR-1224-5p/nuclear receptor binding SET domain protein 2 (NSD2) axis. Bioengineered.

[18] Li Y, Hong X, Zhai J, Liu Y, Li R, Wang X (2023). Novel circular RNA circ-0002727 regulates miR-144-3p/KIF14 pathway to promote lung adenocarcinoma progression. Front Cell Dev Biol.

[19] Mann M, Wright PR, Backofen R (2017). IntaRNA 2.0: enhanced and customizable prediction of RNA-RNA interactions. Nucleic Acids Res.

[20] Agarwal V, Bell GW, Nam JW, Bartel DP (2015). Predicting effective microRNA target sites in mammalian mRNAs. Elife.

[21] Chen Y, Wang X (2020). miRDB: an online database for prediction of functional microRNA targets. Nucleic Acids Res.

[22] Li B, Wu P, Fu W, Xiong Y, Zhang L, Gao Y (2019). The role and mechanism of miRNA-1224 in the polygonatum sibiricum polysaccharide regulation of bone marrow-derived macrophages to osteoclast differentiation. Rejuvenation Res.

[23] Lee JM, Kim MJ, Lee SJ, Kim BG, Choi JY, Lee SM (2021). PDK2 Deficiency prevents ovariectomy-induced bone loss in mice by regulating the RANKL-NFATc1 pathway during osteoclastogenesis. J Bone Miner Res.

[24] Liang B, Burley G, Lin S, Shi YC (2022). Osteoporosis pathogenesis and treatment: existing and emerging avenues. Cell Mol Biol Lett.

[25] Li Z, Li D, Chen R, Gao S, Xu Z, Li N (2023). Cell death regulation: a new way for natural products to treat osteoporosis. Pharmacol Res.

[26] Dong Y, Fan X, Wang Z, Zhang L, Guo S (2020). Circ_HECW2 functions as a miR-30e-5p sponge to regulate LPS-induced endothelial-mesenchymal transition by mediating NEGR1 expression. Brain Res.

[27] Gargano G, Oliviero A, Oliva F, Maffulli N (2021). Small interfering RNAs in tendon homeostasis. Br Med Bull.

[28] Gargano G, Oliva F, Oliviero A, Maffulli N (2022). Small interfering RNAs in the management of human rheumatoid arthritis. Br Med Bull.

[29] Gargano G, Asparago G, Spiezia F, Oliva F, Maffulli N (2023). Small interfering RNAs in the management of human osteoporosis. Br Med Bull.

[30] van Wijnen AJ, van de Peppel J, van Leeuwen JP, Lian JB, Stein GS, Westendorf JJ (2013). MicroRNA functions in osteogenesis and dysfunctions in osteoporosis. Curr Osteoporos Rep.

[31] Singleton Q, Vaibhav K, Braun M, Patel C, Khayrullin A, Mendhe B (2019). Bone marrow derived extracellular vesicles activate osteoclast differentiation in traumatic brain injury induced bone loss. Cells.

[32] Shen J, Hung MC (2015). Signaling-mediated regulation of MicroRNA processing. Cancer Res.

[33] Ganetzky R, McCormick EM, Falk MJ. Primary pyruvate dehydrogenase complex deficiency overview. In: Adam MP, Mirzaa GM, Pagon RA, Wallace SE, Bean LJH, Gripp KW, Amemiya A editors. GeneReviews((R)). Seattle, WA; 1993. 34138529

